# In Situ Neutralization and Detoxification of LPS to Attenuate Hyperinflammation

**DOI:** 10.1002/advs.202302950

**Published:** 2023-07-10

**Authors:** Xiaoyu Li, Shaoqi Qu, Xiangbin Song, Congming Wu, Jianzhong Shen, Kui Zhu

**Affiliations:** ^1^ National Key Laboratory of Veterinary Public Health Security College of Veterinary Medicine China Agricultural University Beijing 100193 China; ^2^ Guangdong Laboratory for Lingnan Modern Agriculture Guangzhou 510642 China

**Keywords:** acyloxyacyl hydrolase, antibiotic, delivery systems, lipopolysaccharide

## Abstract

Hyperinflammation elicited by lipopolysaccharide (LPS) that derives from multidrug‐resistant Gram‐negative pathogens, leads to a sharp increase in mortality globally. However, monotherapies aiming to neutralize LPS often fail to improve the prognosis. Here, an all‐in‐one drug delivery strategy equipped with bactericidal activity, LPS neutralization, and detoxification is shown to recognize, kill pathogens, and attenuate hyperinflammation by abolishing the activation of LPS‐triggered acute inflammatory responses. First, bactericidal colistin results in rapid bacterial killing, and the released LPS is subsequently sequestered. The neutralized LPS is further cleared by acyloxyacyl hydrolase to remove secondary fatty chains and detoxify LPS in situ. Last, such a system shows high efficacy in two mouse infection models challenged with *Pseudomonas aeruginosa*. This approach integrates direct antibacterial activity with in situ LPS neutralizing and detoxifying properties, shedding light on the development of alternative interventions to treat sepsis‐associated infections.

## Graphical abstract

Hyperinflammation elicited by LPS that derives from Gram‐negative pathogens leads to a sharp increase in mortality globally. Monotherapies aiming to anti‐bacteria or anti‐inflammation have failed to improve prognosis. Here, an all‐in‐one drug delivery strategy equipped with bactericidal activity, LPS neutralization, and detoxification is shown to recognize, kill pathogens, and attenuate hyperinflammation by abolishing the activation of LPS‐triggered acute inflammatory responses.

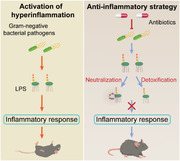

## 1 Introduction

Sepsis is a systemic inflammatory disorder caused by acute microbial infections, particularly Gram‐negative bacteria.^[^ ^1^, ^2^ ^]^ It has been reported that sepsis impacts 31 million people globally and leads to five million deaths annually.^[^ ^3^, ^4^ ^]^ Nowadays, there are no specific therapeutics to treat sepsis due to the complicated immune responses.^[^ ^5^, ^6^ ^]^ Lipopolysaccharide (LPS), a major component in the outer membrane of Gram‐negative bacteria, plays a dominant role in sepsis.^[^ ^7^ ^]^ There are two forms of released LPS from bacteria including LPS monomers and outer membrane vesicles (OMVs).^[^ ^8^ ^]^ LPS monomers trigger the release of pro‐inflammatory cytokines through toll‐like receptors,^[^ ^9^ ^]^ whereas OMVs deliver toxins or effectors into the cytosol, trigger caspase‐11 activation against bacterial infections, and so on.^[^ ^10^ ^]^ Therefore, the development of alternative strategies based on the rapid elimination of bacterial pathogens and subsequent timely interventions of LPS activation are urgently needed.

There are no direct treatments for sepsis. Current approaches are mainly symptomatic supportive (e.g., broad‐spectrum antibiotics, intravenous fluids, and surgeries).^[^ ^3^, ^11^ ^]^ Antibodies either targeting LPS or neutralizing specific cytokines such as tumor necrosis factor‐alpha (TNF‐*α*) and interleukin‐6 (IL‐6) have provided unsatisfactory treatments for sepsis.^[^ ^12^, ^13^ ^]^ In addition, the capture and neutralization of histone, a major inflammatory mediator during sepsis, serve as a potential molecular target for sepsis.^[^ ^14^, ^15^ ^]^ However, these considerable efforts are almost failing to improve the symptoms of sepsis.^[^ ^16^ ^]^ Because the undetoxified and aetiological LPS will persistently aggravate the prognosis.

Neutralizing LPS in situ represents an alternative intervention to fundamentally abolish or ameliorate LPS‐induced inflammatory responses. The LPS‐binding molecules/polymers include both small molecules (peptides) and macromolecules (proteins and nanoparticles).^[^ ^17^, ^18^, ^19^ ^]^ Compared to macromolecules, cationic antimicrobial peptides such as colistin and chondroitin have simple structures with potent anti‐inflammatory activities.^[^ ^4^, ^20^, ^21^ ^]^ However, the reversible binding between LPS and such molecules still constitutes a potential risk. How to detoxify the free and neutralized LPS remains quite challenging, particularly in vivo. It has been demonstrated that LPS can be detoxified directly through acyloxyacyl hydrolase (AOAH) dependent enzymatic degradation by several phagocytic cell types and lysosome‐mediated degradation by macrophages or dendritic cells.^[^ ^22^, ^23^ ^]^ Thus, we hypothesized that LPS neutralization combined with LPS detoxification would greatly benefit the treatment of sepsis.

**Scheme 1:**
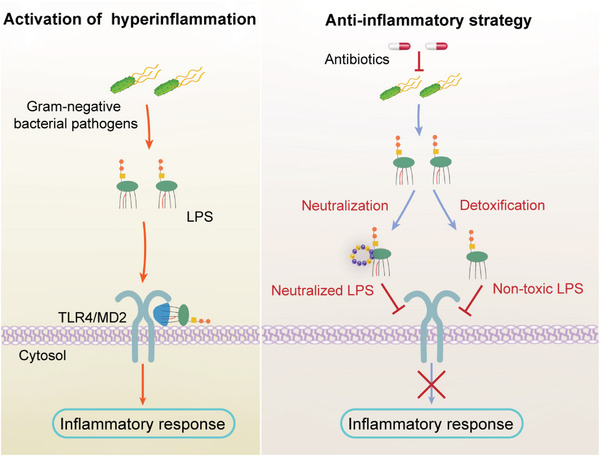
Schematic illustration of blocking LPS activation for anti‐inflammatory therapy. Lipopolysaccharide (LPS) released from Gram‐negative bacteria binds to Toll‐like receptor 4 (TLR4)‐MD2 complex and activates nuclear factor‐*κ*B (NF‐*κ*B) pathway to produce cytokines or prostaglandin (left part). The combination of anti‐bacteria and in situ LPS neutralization attenuates hyperinflammation by blocking cytokines cascades (right part). Bactericidal agents kill Gram‐negative pathogens to release LPS which are timely neutralized through binding to LPS to form neutralized‐LPS. Then, the neutralized or free LPS are detoxified by hydrolyzing the secondary acyl chains of Lipid A, rendering it immunologically inert. The approach with both antibacterial and anti‐inflammatory properties provides a guideline to improve efficacy in sepsis.

In this work, we proposed an all‐in‐one strategy with simultaneous antibacterial and anti‐inflammatory effects to attenuate sepsis by blocking the cascaded stages of LPS activation (**Scheme** **1**). Bactericidal antibiotics target Gram‐negative bacterial pathogens to release LPS, which is subsequently neutralized and degraded to abolish the following inflammatory response. These results provide a new intervention strategy for the treatment of sepsis through ameliorating hyperinflammation‐associated infections in clinics.

## 2 Results

### 2.1 Neutralization of Monomeric LPS and OMVs

**Figure 1:**
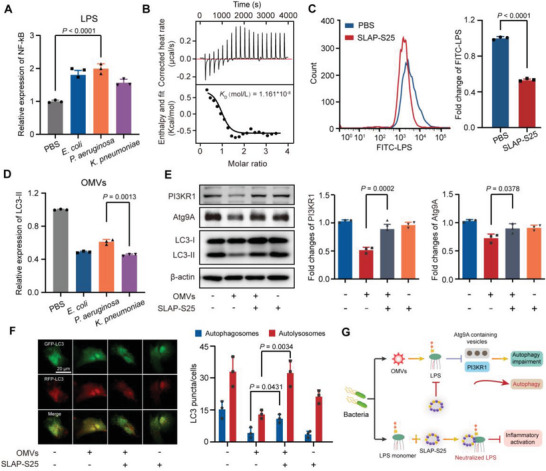
Blocking LPS activation to attenuate inflammation and restore autophagy. A) Relative expression of NF‐*κ*B in mouse alveolar macrophages (MH‐S) in the presence of LPS (1 µg mL^−1^) derived from diverse bacteria (*E. coli*, *P. aeruginosa*, and *K. pneumoniae*). B) The affinity between SLAP‐S25 and LPS derived from *P. aeruginosa* was analyzed by isothermal titration calorimetry. C) LPS accumulation in MH‐S cells after being treated with SLAP‐S25 based on flow cytometry analysis. D) Relative expression of autophagy marker LC3‐II in the presence of OMVs derived from different bacteria (*E. coli*, *P. aeruginosa*, and *K. pneumoniae*). E) Relative expression and quantification of autophagy relative protein PI3KR1, Atg9A, and LC3 in A549 cells. F) Representative images of LC3 staining in A549 cells infected with GFP‐RFP‐LC3 adenovirus. Green (GFP), red (autolysosomes), and yellow (merged, autophagosome). Scale bar = 20 µm. G) Scheme of SLAP‐S25 neutralized OMVs and LPS monomers. Experiments in A, C, D, E and F were performed as three biologically independent experiments, and the mean ± s.d. is shown, *n* = 3. *P* values were determined using One‐way ANOVA test.

LPS monomers are released after the lysis of Gram‐negative bacterial pathogens killed by antibiotics. We first assessed the inflammatory response of host cells treated with LPS derived from *Escherichia coli*, *Pseudomonas aeruginosa*, and *Klebsiella pneumoniae*, based on the canonical nuclear factor‐*κ*B (NF‐*κ*B) pathway.^[^ ^24^ ^]^ The results showed that LPS caused an inflammatory response in mouse alveolar macrophages (MH‐S). Notably, LPS derived from *P. aeruginosa* exhibited sharp upregulation of NF‐*κ*B, TNF‐*α* and IL‐6 expression and activated inflammation (**Figure** **1**A; Figure S1, Supporting Information). Interestingly, the pretreatment of macrophages with SLAP‐S25,^[^ ^25^, ^26^, ^27^ ^]^ an antibiotic adjuvant with the capability to target LPS, reduced the inflammatory response triggered by LPS derived from *E. coli* and *P. aeruginosa* (Figure 1B; Figure S1 and Table S1, Supporting Information,). Next, we investigated the efficiency of SLAP‐S25 neutralizing LPS in macrophages based on flow cytometry analysis, and found the decreased binding and internalization of LPS (Figure 1C). It is indirectly proved the neutralization of extracellular free LPS. It consists with the previous report that monomeric LPS molecules are prerequisite for the formation of endotoxin aggregates, to cause subsequent biological activities.^[^ ^28^ ^]^ These findings demonstrate that the neutralization of monomeric LPS blocks the activation of inflammation.

The other form of LPS is OMVs, which play a critical role in the delivery of cytosolic LPS and activates a protein called caspase‐11 that triggers inflammation and cell death.^[^ ^8^ ^]^ We compared the expression of LC3‐II to characterize cell function induced by OMVs from different bacteria (*E. coli*, *P. aeruginosa*, and *K. pneumoniae*). We found more severe cell function loss after treatment with OMVs from *K. pneumonia* compared with *P. aeruginosa* (Figure 1D), which agreed with previous observations that *K. pneumonia*‐derived LPS induced high expression levels of TNF‐*α* and IL‐6 in MH‐S cells (Figure S1B,C, Supporting Information). Meanwhile, OMV‐induced cytotoxicity was attenuated by the supplementation of SLAP‐S25 (Figure S2A, Supporting Information). We then sought to explore the underlying mechanism of SLAP‐S25 neutralizing cytosolic LPS. We observed increased expression levels of autophagy‐related proteins PI3KR1, Atg9A, and LC3‐II, suggesting that inhibited autophagy in the presence of OMVs is restored (Figure 1E; Figure S2B, Supporting Information). Correspondingly, autophagosomes and autolysosomes increased in A549 cells treated with OMVs in the presence of SLAP‐S25 (Figure 1F). These findings suggest that neutralized OMVs or LPS monomers attenuate inflammatory responses (Figure 1G). However, these non‐covalent LPS still have the potential to provoke inflammation.

### 2.2 LPS Detoxification by AOAH

**Figure 2:**
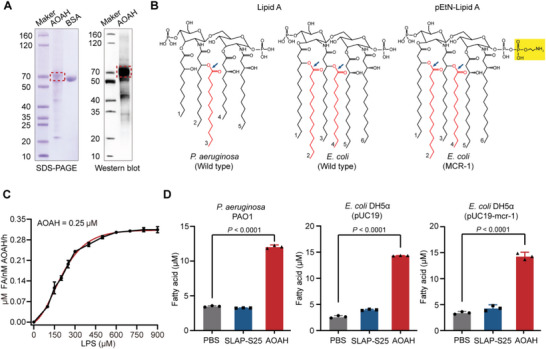
Detoxication of LPS by lipase AOAH. A) Purified recombinant AOAH was verified based on SDS‐PAGE (left) and western blotting (right). Bands of AOAH are depicted by dotted lines. B) Structures of the lipid A moiety in *P. aeruginosa* and *E. coli*. Secondary chains in red are removed by AOAH, the broken bonds are marked by blue arrows and phosphoethanolamine is indicated in yellow. C) The activity of AOAH against different concentrations of LPS in *P. aeruginosa*. D) Total fatty acid amounts of LPS derived from diverse bacteria *P. aeruginosa* PAO1, *E. coli* DH5*α* (pUC19), and *E. coli* DH5*α* (pUC19‐mcr‐1) hydrolyzed by AOAH. Data represent three biological replicates. *P*‐values were determined using One‐way ANOVA test.

To thoroughly abolish the toxicity of free (not neutralized) or sequestered LPS, it is better to eliminate the intact structure of LPS. Previous studies demonstrate that a unique lipase, AOAH, deacylated the secondary fatty acid chain from lipid A of LPS to inactive forms, to exterminate bioactive LPS.^[^ ^29^, ^30^, ^31^ ^]^ We constructed and purified recombinant AOAH from *E. coli*‐based expression systems (**Figure** **2**A). Although LPS derived from bacteria such as *P. aeruginosa* and *E. coli* showed various patterns (Figure 2B), we found that AOAH showed general enzymatic activity to remove fatty acyl chains from the lipid A moieties based on a free fatty acid fluorometric assay (Figure 2C). Markedly, fewer fatty acids were produced in *P. aeruginosa* because these are penta‐acyl and hexa‐acyl lipid A moieties in *P. aeruginosa* and *E. coli*, respectively (Figure 2D). Some bacteria can modify their LPS structure to evade the host immune system or adapt to different environments.^[^ ^32^, ^33^, ^34^ ^]^ Intriguingly, AOAH has an equal deacylation on either wild‐type or phosphoethanolamine‐modified LPS, mediated by *mcr* genes (Figure 2D). Collectively, it indicates that the robust deacylation activity of AOAH is a promising component for paralyzing LPS.

### 2.3 All‐in‐One Approach to Target Bacteria

**Figure 3:**
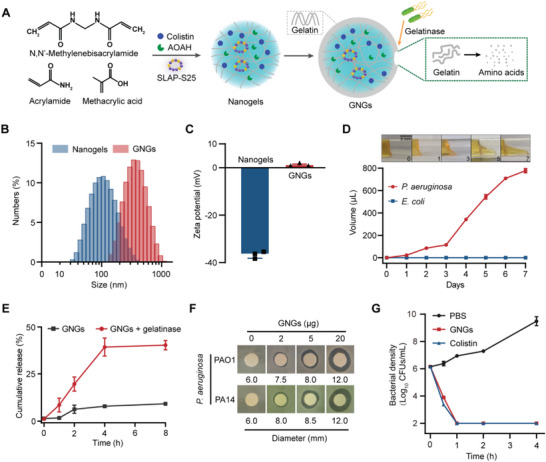
Characterization of all‐in‐one platform with robust antibacterial activity. A) Schematic of the preparation and response process of the GNGs. B,C) The average diameter (B) and surface zeta potential (C) of nanogels and GNGs were confirmed by dynamic light scattering. D) Gelatinase hydrolysis experiment of *P. aeruginosa*‐derived gelatinase hydrolyzed gelatin in 7 d. *E. coli* ATCC 25922 as a negative control. Scale bar = 5 mm. E) Cumulative release of FITC from GNGs with or without gelatinase secreted by *P. aeruginosa* PAO1. F) Inhibition zones of GNGs against *P. aeruginosa* PAO1 and PA14 for 12 h. G) Time‐kill kinetics of *P. aeruginosa* PAO1 at the exponential phase treated with GNGs or colistin. Experiments in (B), (C), (E), and (G) were performed as three biologically independent experiments, and the mean ± s.d. is shown, *n* = 3. Data in (D) and (F) represent two biological replicates. *P*‐values were determined using One‐way ANOVA test.

To demonstrate the proof of concept of simultaneous antibacterial and anti‐inflammatory action, we integrate the functions of initial recognition, killing, and elimination of Gram‐negative bacterial pathogens. Therefore, we constructed an all‐in‐one platform named gelatin‐coated nanogels (GNGs), loading them with bactericidal colistin, SLAP‐S25 neutralizing LPS, and AOAH degrading LPS, using the notorious *P. aeruginosa* as a model. The preparation processes of GNGs were divided into two parts: nanogels loaded with cargoes and responsive gelatin coated on the nanogels (**Figure** **3**A). Nanogels were prepared with the biodegradable poly (acrylamideco‐methacrylic acid) [P(AAm‐co‐MAA)] copolymers, according to a previously optimized inverse emulsion polymerization method.^[^ ^35^ ^]^ GNGs with uniform particle sizes and a net negative charge were obtained based on dynamic light scattering analysis (Figure 3B,C). GNGs have an average diameter of approximately 373.3 ± 24.7 nm, which is slightly larger than the uncoated nanogels (Figure 3B). Meanwhile, GNGs exhibited a strongly reversed surface charge of approximately 1.203 ± 0.317 mV (Figure 3C), indicating the successful coating of gelatin.

Next, to verify the specific recognition of GNGs, we performed a gelatin hydrolysis experiment to assess the capability of *P. aeruginosa* to liquefy gelatin. The gelatinase produced by *P. aeruginosa* liquefied gelatin into a flowable liquid (Figure 3D), indicating the feasibility of responsive release. Consistently, the cumulative release profile of indicator FITC increased from 9.11% to 40.2% in the presence of gelatinase from *P. aeruginosa* (Figure 3E). Lastly, to verify the functional feasibility of GNGs, the antibacterial efficiency was characterized by inhibition zone and time‐kill kinetics. The results showed the concentration‐dependent and rapid bactericidal activity of GNGs against *P. aeruginosa* PAO1 and PA14 (Figure 3F,G; Figure S3A, Supporting Information). Particularly, *P. aeruginosa* biofilm formation, which is associated with chronic infections and antibiotic resistance, was significantly inhibited by GNGs (Figure S3B, Supporting Information). Collectively, these results demonstrate that GNGs provide a precision response to combat gelatinase‐positive bacterial pathogens.

### 2.4 Dynamics of LPS Inactivation

**Figure 4:**
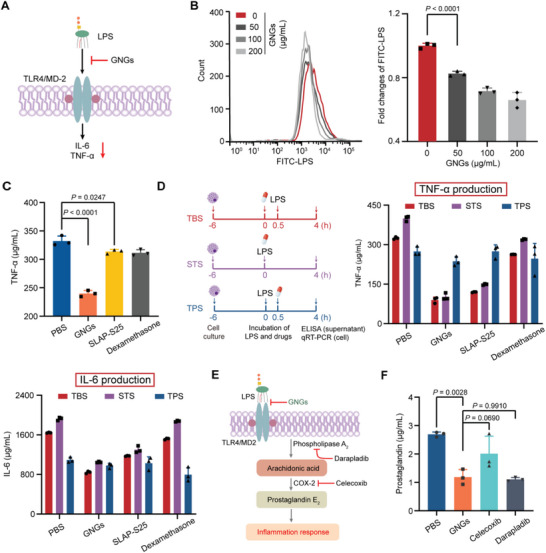
Blocking the initial step of LPS activation. A) Scheme of LPS neutralized by GNGs. B) LPS accumulation in MH‐S cells after being treated with GNGs based on flow cytometry analysis. C) Relative production of TNF‐*α* in MH‐S cells in the presence of LPS. MH‐S cells were treated with different compounds for 30 min, washed with PBS three times to remove free drugs and then stimulated with LPS for 4 h. D) The scheme shows the experimental design. Relative production (supernatant) and expression (cell) of TNF‐*α* and IL‐6 with the treatments added before (TBS), simultaneous (STS) or after (TPS) the stimulation of LPS in MH‐S cells, respectively. E) Schematic illustration of anti‐inflammatory mechanisms among GNGs and non‐steroidal anti‐inflammatory agents. F) Relative production of prostaglandin E_2_ in MH‐S cells in the presence of LPS with different treatments by high performance liquid chromatography (HPLC) analysis. Experiments in (B), (C), (D), and (F) were performed as three biologically independent experiments, and the mean ± s.d. is shown, *n* = 3. *P*‐values were determined using One‐way ANOVA test.

Given that GNGs provide robust antibacterial activity, we further explored the anti‐inflammatory function of GNGs. To explore the dynamic of LPS inactivation, the production of proinflammatory cytokines TNF‐*α* and IL‐6 was assessed (**Figure** **4**A). On the one hand, we evaluated the efficiency of GNGs in neutralizing LPS in macrophages based on flow cytometry analysis and found that GNGs reduced the binding and internalization of LPS with the host cells in a dose‐dependent manner (Figure 4B). On the other hand, we observed that macrophages activated by the previous treatment with GNGs maintained their anti‐inflammatory activities (Figure 4C). These data may suggest that the anti‐inflammatory effects of GNGs are not only dependent on LPS neutralization via direct binding but also rely on interactions with the host cells. Therefore, we determined the ability of GNGs treatment before LPS stimulation (TBS), simultaneous treatment with LPS stimulation (STS), and treatment post LPS stimulation (TPS), and found that GNGs attenuate the expression and production of either TNF‐*α* and IL‐6 in TBS and STS (Figure 4D; Figure S4, Supporting Information). These results indicate that both simultaneous and previous treatments with GNGs effectively block the proinflammatory signaling cascades in macrophages. It demonstrates that the inhibition of LPS activation is a more efficient therapeutic strategy than blocking the activated inflammatory response.

Compared to the classic non‐steroidal anti‐inflammatory drugs that block the synthesis of prostaglandin E_2_ to reduce inflammation,^[^ ^36^ ^]^ we evaluated the downstream inflammatory products in the presence of GNGs and non‐steroidal anti‐inflammatory drugs (celecoxib targeting COX‐2 and darapladib inhibiting phospholipase A_2_, Figure 4E). We observed similar levels of prostaglandin production based on high‐performance liquid chromatography (HPLC) analysis (Figure 4F; Figure S5, Supporting Information), consistent with the fact that free LPS is a prerequisite for eliciting inflammation.^[^ ^37^ ^]^ Collectively, our findings indicated that GNGs efficiently attenuate LPS‐induced inflammation by neutralizing LPS, suggesting that the upstream inhibition of information is a promising approach to combating sepsis.

### 2.5 Efficacy in Mouse Infection Models

**Figure 5:**
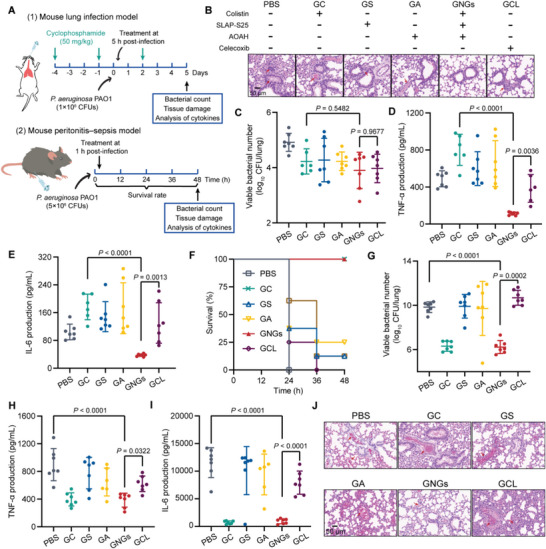
Robust antibacterial and anti‐inflammatory activity in mouse infection models. A) The experimental protocol of mouse lung infection and peritonitis‐sepsis model. B–E) Representative hematoxylin and eosin‐stained lungs (B) of mice in the lung infection model, together with bacterial counts (C), the production of TNF‐*α* (D), and the production of IL‐6 (E) in the infected lungs. Celecoxib group as the positive group. F) Survival rates of mice in the peritonitis‐sepsis model (*n* = 8). G–I) Bacterial loads (G), the production of TNF‐*α* (H) and IL‐6 (I) in the lungs of mice in the peritonitis‐sepsis model (*n* = 7). J) Representative hematoxylin and eosin‐stained lung histology sections of mice in the peritonitis‐sepsis model. Scale bar = 50 µm. Data presented as mean ± s.d. *P*‐values were determined using One‐way ANOVA test.

Given the attractive biological activity of GNGs in vitro, we first performed cell cytotoxicity (RAW 264.7, MH‐S, Vero, and HepG2 cell lines) and hemolytic activity in sheep red cells to evaluate the biocompatibility of GNGs before in vivo evaluation. GNGs showed low cytotoxicity to tested mammalian cells and low hemolysis as well, although GNGs showed 20% cytotoxicity at the experimental concentration in MH‐S cells (Figure S6, Supporting Information). These results suggest that GNGs is a potent and safe therapeutic approach in vivo. Therefore, we investigated its therapeutic potential in a lung infection model and a peritonitis‐sepsis model in mice infected with *P. aeruginosa* PAO1 (**Figure** **5**A). In the mouse lung infection model, GNGs alleviated the pathological damage effectively (Figure 5B; Figure S7A, Supporting Information). Meanwhile, bacterial loads in the lungs of mice significantly reduced under either GNGs or colistin therapies at 48 h (Figure 5C; Figure S7B, Supporting Information). Remarkably, GNGs showed better anti‐inflammatory activities than colistin (GC), as demonstrated by the fact that the productions of TNF‐*α* and IL‐6 in the presence of GNGs were approximately fourfold lower than those in the GC group (Figure 5D,E; Figure S7C–E, Supporting Information). Furthermore, we established an acute mouse peritonitis‐sepsis model to assess the efficacy of GNGs in systemic infections. All mice survived after treatment with GNGs (6 mg kg^−1^) within 48 h, superior to the PBS group (all died within 24 h) (Figure 5F; Figure S8A, Supporting Information). Consistently, the bacterial loads and cytokines in major organs were significantly reduced in the presence of GNGs and colistin (Figure 5G–I; Figures S8B–F and S9, Supporting Information). Compared to the solely used colistin, GNGs demonstrate comparable antibacterial and anti‐inflammatory activities against *P. aeruginosa*, suggesting that GNGs may be more suitable for infections where inflammation is the main symptom. Meanwhile, GNGs ameliorate the pathological damages such as decreased hemorrhage and congestion in the lungs of severe septic mice (Figure 5J; Figure S10, Supporting Information). Altogether, these results demonstrate the potential of GNGs as a platform to treat sepsis through an all‐in‐one approach.

## 3 Discussion

In Gram‐negative pathogen‐associated infections, the neutralization of LPS and regulation of the immune response are as important as the direct antibacterial effect.^[^ ^38^ ^]^ Thus, we constructed an all‐in‐one nanoplatform to kill bacterial pathogens and paralyze LPS‐induced inflammatory responses. The LPS released from Gram‐negative bacteria by bactericidal antibiotics is subsequently neutralized and detoxified to initially inhibit inflammation. This multifunctional platform sheds light on alternative strategies to treat clinical sepsis‐related inflammation.

LPS comprises three functional regions, including the lipid A moiety, the core oligosaccharide, and the O‐polysaccharide (O‐antigen).^[^ ^39^ ^]^ A long polysaccharide chain is highly associated with the formation of stable aggregates and extended residence time in plasma, which trigger inflammatory potential.^[^ ^40^ ^]^ Lipid A, together with the core oligosaccharide, plays a crucial role in LPS‐induced inflammation, but O‐antigen is a non‐essential part of inflammation. Lipid A represents the conserved, essential, proinflammatory moiety of LPS molecules.^[^ ^41^ ^]^ Both the number of fatty acid chains and chemically modified phosphate groups in lipid A modulate the inflammation response. Normally, the activation of LPS‐related inflammation requires the presence of hexa‐acyl chains in lipid A, whereas the efficiency of hydrophobic analogs with penta‐acyl and tetra‐lipid chains mitigates more than 100‐fold and completely loses activity by structure determination, respectively.^[^ ^42^, ^43^ ^]^ Interestingly, our results show that *P. aeruginosa*‐derived LPS with penta‐acyl chains caused a more severe inflammatory response than the LPS carrying hexa‐acyl chains in *E. coli*. Nevertheless, it should be noted that whether the length of the core oligosaccharide or other accessories contributed to such a phenomenon remains elusive. For example, the phosphoethanolamine‐modified 1, 4′ phosphate groups of lipid A dramatically increase mortality in mice infected with *E. coli* due to acute inflammation.^[^ ^44^ ^]^ Notably, the acyl chains from various bacteria and the modified phosphate group or not have no effect on the selective hydrolysis of ester bonds in the acyloxyacyl‐linked chains by AOAH. However, the affinity between different LPS and AOAH need further exploration.

Multiple signal transduction pathogenic pathways in sepsis are potential classic targets to improve treatments and prognoses. First, inflammatory mediators such as cytokines and prostaglandins at the late stage of sepsis can be directly alleviated by routinely used anti‐inflammatory agents, including non‐steroidal anti‐inflammatory drugs and glucocorticoids.^[^ ^45^, ^46^ ^]^ Apparently, such supportive therapies may promptly have the desired effect, whereas the pre‐existing etiological agents, particularly LPS, may cause persistent and recurrent inflammation. Therefore, pioneering works that block inflammatory caspases in earlier steps by inhibiting gasdermin D preformation in inflammasome pathways,^[^ ^47^ ^]^ and by disrupting the binding of high mobility group box 1 protein and LPS,^[^ ^48^ ^]^ thereby preventing cytokines release and pyroptosis to counteract inflammation. Nevertheless, the more dominant pro‐inflammatory pathway of LPS associated sepsis has been continually neglected, because the activation of the TLR4‐MD2 receptor by LPS initiates the inflammatory caspases. Hence, the promising strategy that neutralizes LPS in suits has attracted more attention recently.^[^ ^49^, ^50^ ^]^ Given that noncovalent neutralization or binding of LPS are suffering from the low affinity of the modified phosphate group of Lipid A, encoded by the emerging *mcr* genes.^[^ ^51^, ^52^ ^]^ We propose an alternative strategy that integrates anti‐bacteria and anti‐inflammation to recognize, neutralize, and detoxify LPS to reduce the activation of sepsis and originally inhibit the possibility of inflammation. Remarkably, the smart delivery system loads multiple antibiotics and biological recognition elements of interest with high specificity for variable pathogens. How the dynamic of our established system eliminates the residual LPS is not the focus of this current work, but will be probed in the following studies.

In summary, we have proven that combating sepsis in a stepwise manner greatly benefits the treatment of hyperinflammation. Gram‐negative bacterial pathogens are killed by bactericides to release LPS, which is subsequently neutralized and detoxified to attenuate the excessive inflammation. Our strategy indicates that the combined antibacterial and anti‐inflammatory effects are a promising solution to the perfect adaptation of innate immunity against sepsis.

## 4 Experimental Section

### Bacteria

Both *E. coli* (ATCC 25922) and *K. pneumoniae* (ATCC 43816) used in this study were obtained from the American Type Culture Collection (ATCC). *P. aeruginosa* PAO1 and PA14 strains were used in this study, which have been previously characterized.^[^ ^53^ ^]^

### Cell Lines and Cell Culture

Mouse monocyte‐macrophage leukemia cells (RAW264.7) and mouse alveolar macrophage cells (MH‐S) were cultured in Roswell Park Memorial Institute (RPMI) 1640 (Gibco) supplemented with 10% heat‐inactivated FBS (Invitrogen) and 1% (w:v) penicillin‐streptomycin at 37 °C in a 5% CO_2_ atmosphere. African green monkey kidney cells (Vero) and Human hepatocellular carcinomas (HepG2) were cultured in Dulbecco's modified Eagle medium (DMEM) (Gibco) supplemented with 10% heat‐inactivated FBS (Invitrogen) and 1% (w:v) penicillin‐streptomycin at 37 °C in a 5% CO_2_ atmosphere. All cell lines were obtained from the ATCC.

### Mice

Female C57BL/6 aged 6–8 weeks were obtained from the Beijing Vital River Laboratory. Mice were adapted to standardized environmental conditions (temperature = 23 ± 2 °C; humidity = 55 ± 10%) for 1 week before infection. Mice were maintained in strict accordance with the regulations for the Administration of Affairs Concerning Experimental Animals approved by the State Council of the People's Republic of China (11‐14‐1988). The animal study protocols were performed in accordance with the relevant guidelines and regulations (ID: SKLAB‐B‐2010‐003). The laboratory animal usage license number is SYXK‐2016‐0008, certified by the Beijing Association for Science and Technology.

### Gelatin‐Coated Nanogels Synthesis

Nanogels were prepared by an inverse emulsion polymerization method, according to the previous study with appropriate modifications.^[^ ^35^ ^]^ Acrylamide (0.356 g), methacrylic acid (94 µL), and *N, N’*‐methylenebisacrylamide (0.111 g) were dissolved in ultrapure water (1.563 mL), along with 25 µL of *N, N, N'N’*‐tetramethylethylenediamine. The solution was sonicated for 10 min to ensure a uniform aqueous phase. Surfactants of dioctyl sulfosuccinate (AOT, 0.383 g) and Brij‐30 (1.73 mL) were dissolved in 30 mL of hexane to form the organic phase. The organic phase was stirred rapidly until all of the surfactants dissolved. The aqueous phase was then added to the organic phase to form the prepolymer emulsion. Following 30 min of nitrogen purging to remove dissolved oxygen, an initiator solution of ammonium persulfate (APS, 100 mg mL^−1^) in ultrapure water was purged with nitrogen for 10 min, and 50 µL of the initiator solution was injected into the prepolymer emulsion. Reactions were allowed to proceed with constant stirring at room temperature for 2 h. Then an equal volume of ethanol was added and centrifuged at 3200 *g* for 5 min to precipitate synthesized nanoparticles.

Precipitation was repeated two additional times in 50 mL of ethanol prior to further purification. Nanogels were resuspended in 15 mL of 0.5 m sodium hydroxide solution and 35 mL of acetone and centrifuged at 3200 *g* for 5 min. Then nanogels were dialyzed against ultrapure water for 48 h to remove unreacted reactants and lyophilized to obtain a white nanoparticle powder.

To fabricate the GNGs, gelatin layers were coated onto the nanogels using a simple mixing method based on electrostatic interaction. Nanogels and gelatin were co‐incubated for 30 min and centrifuged at 10 000 rpm for 20 min. Nanogels loaded with SLAP‐S25, colistin, and AOAH were added to the nanogel solution before the addition of the gelatin coating. In the following works, GNGs were coated with gelatin to target bacteria, whereas GNGs without a gelatin coating were used for LPS treatment.

### Size and Surface Zeta Potential Analysis

To test the size and surface zeta potential of nanogels and GNGs, the nanoparticles were dispersed into water and sonicated for 10 min to ensure a uniform dispersion. The resulting dispersions were measured by dynamic light scattering using the Malvern Zetasizer Nano ZS90 (Malvern, UK).

### Gelatin Liquefaction Test

The gelatin medium was autoclaved at 115 °C for 20 min and packed in 5 mL of every tube. *P. aeruginosa* PAO1 (2 × 10^9^ CFUs) was inoculated in gelatin medium and stationary cultured at 22 °C for 7 d. The volume of liquefied gelatin was quantified by a micropipette. *E. coli* ATCC 25922 as a negative control.

### In Vitro Release Tests

The cumulative release of FITC in vitro from the GNGs (25 mg) in 80 mL of PBS (pH 7.2) was carried out by dialyzing at 300 rpm for 8 h. At the predetermined time intervals (0, 1, 2, 4, and 8 h), the medium was withdrawn in 0.2 mL (three repeats) and replaced with an equal volume of PBS. The fluorescence of samples was measured using an excitation wavelength of 490 nm and an emission wavelength of 525 nm with the Infinite M200 Microplate Reader (Tecan).

### Disk Diffusion Assay

The antibacterial activity of GNGs was determined using the disk diffusion assay, following the Clinical Laboratory Standards Institute 2021 guideline.^[^ ^54^ ^]^ Mueller–Hinton agar (MHA) plate containing the medium that had been inoculated with a suspension of bacteria (1 × 10^7^ CFUs) to give confluent growth. Inhibitor disks were made by adding the indicated quality of GNGs to the thick Whatman filter paper (6 mm) disk. The plates were placed in the 37 °C incubators for 12 h. The inhibition zones were determined by measuring the diameter of the no‐growth zone. A 6 mm zone of inhibition indicates no inhibition.

### Time–Kill Assay

Time–kill assays were performed in tubes containing bacteria at an exponential phase. The bacteria were treated with drugs (twofold MIC) and incubated at 37 °C for the indicated times. Then 100 µL bacterial suspensions with tenfold serial dilutions were plated onto tryptic soy agar (TSA) plates and incubated at 37 °C for 18 h. Colonies between 30 and 300 were counted, and CFUs per milliliter were calculated.

### Biofilm Inhibition Assay

The biofilm inhibition assay was performed in a clear 96‐well microtiter plate. GNGs were twofold diluted in Luria–Bertani broth (LB) and mixed with an equal volume of bacterial suspensions in LB containing ≈1.5 × 10^6^ CFUs mL^−1^ in 96‐well microtiter. After 24 h incubation, the planktonic bacteria were removed by washing three times with PBS (pH 7.2) solution. After that, methanol (99%) was added and fixed for 15 min. Then, the plates were allowed to dry and stained with 125 µL 0.1% crystal violet for 5 min. The excess stain was gently rinsed off with tap water. The stain was resolubilized in 95% ethanol, and the absorbance was measured at 600 nm.

### AOAH Expression and Purification

Protein expression and purification were constructed by MerryBio Co.,Ltd (Nanjing, China). Recombinant AOAH was expressed as a secreted protein in mammalian cell HEK293 infected with *E. coli* DH5*α*. The endogenous signal peptide is a C‐his tag. AOAH was isolated from the expression culture medium using nickel‐iminodiacetic acid (Ni‐IDA) resin (MerryBio Co.,Ltd), further purified by dialysis treatment in buffer (1×PBS, 5% Glycerol (pH 7.4)), collected by 0.22 µm filter, and concentrated to 1 mm.

### Free Fatty Acid Assay

According to the previous study, the enzymatic activity of AOAH was measured by quantifying the release of fatty acids from the LPS substrate using the free fatty acid fluorometric assay kit.^[^ ^31^ ^]^ Bacteria from exponentially growing cultures were washed three times by AOAH buffer (100 mm NaCl, 20 mm Tris·HCl (pH 7.5)) and adjusted to an OD_600_ equal to 0.5, subsequently incubated with colistin (100 µg mL^−1^) for 24 h and centrifuged at 10 000 rpm for 15 min by an ultrafiltration tube (5 kD). SLAP‐S25 (500 µg mL^−1^), AOAH (5 µm), and GNGs (500 µg mL^−1^) were mixed with re‐dissolved LPS on the ultrafiltration tube membrane by AOAH buffer at 37 °C for 1 h. Then free fatty acids were quantified by a free fatty acid fluorometric assay kit (Cayman Chemical) following the instructions.

### Isothermal Titration Calorimetry (ITC) Spectroscopy

The affinity between SLAP‐S25 and LPS derived from *P. aeruginosa* was characterized by Affinity ITC (TA Instruments) at 25 °C; both 1 mmol L^−1^ LPS and 0.1 mmol L^−1^ SLAP‐S25 were dissolved in ultrapure water. Sequential injections of LPS into the calorimetric cell filled with SLAP‐S25 were repeated 20 times with equilibration intervals of 200 s. The obtained data were processed using the software with the instrument to calculate the equilibrium dissociation constant (*K*_D_).

### Flow Cytometry

MH‐S cells were incubated with GNGs (50, 100, and 200 µg mL^−1^) or SLAP‐S25 (100 µg mL^−1^) at 37 °C for 30 min. Then, FITC‐LPS (100 µg mL^−1^, LPS from *P. aeruginosa*) was added, and the mixture was incubated at 37 °C for another 1 h. After washing MH‐S cells three times with PBS, the cells were collected, and the fluorescent signals of FITC‐LPS in the cells were detected using flow cytometry.

### Safety Assessment

Cytotoxicity was determined using four mammalian cells including RAW 264.7, MH‐S, Vero, and HepG2. Briefly, RAW 264.7 and MH‐S cells were seeded in 1% 1640 at a concentration of 3.5 × 10^4^ cells per well, and Vero and HepG2 cells were seeded in 1% DMEM at a concentration of 1 × 10^4^ cells per well in 96‐well plates. After 6 h of incubation, a fresh medium with GNGs at different concentrations was added and incubated for another 24 h. Cell viability was determined by the LDH cytotoxicity assay kit following the instructions (Beyotime Biotechnology Co., Shanghai, China).

The hemolytic activity and hemolytic rate of GNGs were determined according to the previous report.^[^ ^25^ ^]^ Briefly, sheep blood cells were prepared from fresh, sterile, defibrinated, sheep blood (Beijing Land Bridge Technology) and treated with various concentrations of GNGs ranging from 1 to 200 µg mL^−1^ at 37 °C for 1 h. The absorption of released hemoglobin was measured at 576 nm by an Infinite M200 Microplate reader (Tecan).

### Enzyme‐Linked Immunosorbent Assay (ELISA)

MH‐S cells were plated in 12‐well plates at a density of 7.5 × 10^5^ cells per well. Cells were treated with 50 µg mL^−1^ of SLAP‐S25, GNGs, and 10 µm dexamethasone for 30 min before the final concentration of LPS (1 µg mL^−1^) was added to cell cultures. After 4 h of incubation, the supernatants were obtained for TNF‐*α* and IL‐6 production analysis using ELISA MAX Standard Set Mouse TNF‐*α* and ELISA MAX Standard Set Mouse IL‐6 (Biolegend, US).

The expression of TNF‐*α* and IL‐6 relative to *β*‐actin was detected by qRT‐PCR tests with the PowerUp SYBR Green Kit (Applied Biosystems). Thermal cycling was performed using a two‐step PCR amplification standard procedure at 95 °C for 30 s and 40 cycles of 60 °C for 30 s and 72 °C for 30 s. The qRT‐PCR test was performed using the ABI Quantstudio 7 detection system (Applied Biosystems). The fold changes of gene expression were determined using the 2^−ΔΔCt^ method.

### High‐Performance Liquid Chromatography (HPLC) Analysis

The production of prostaglandin *E*_2_ was quantified by HPLC. The chromatographic separation was carried out on an ODS C18 column. The mobile phase A was acetonitrile, and the mobile phase B was 0.02 m potassium dihydrogen phosphate. The column temperature was 30 °C and the low rate was 1 mL min^−1^. A sample solution of 20 µL was injected into the HPLC system and detected at a wavelength of 196 nm. MH‐S cells were plated in 6‐well plates at a density of 1.5 × 10^6^ cells per well. Cells were treated with SLAP‐S25 (50 µg mL^−1^), GNGs (50 µg mL^−1^), celecoxib (5 µg mL^−1^), and darapladib (1 µg mL^−1^) for 30 min before the final concentration of LPS (1 µg mL^−1^) was added to cell cultures. After 24 h incubation, the supernatants were obtained for prostaglandin quantification.

### Confocal Laser Scanning Microscopy Analysis

Confocal analysis was applied to view the location of LPS from *K. pneumoniae* or OMVs and the change of autophagosome/autophagolysosome induced by OMVs (200 µg mL^−1^) and/or SLAP‐S25 (20 µg mL^−1^). A549 cells were seeded in 1% DMED/F‐12 at a concentration of 3 × 10^4^ cells per well in 24‐well plates. Cells were infected with *K. pneumoniae* (multiplicity of infection (MOI) = 20) or OMVs (200 µg mL^−1^) for 6 h, fixed in 4% paraformaldehyde, 0.1% Triton X‐100 permeabilized for 15 min, and incubated with 2% BSA at room temperature for 1 h. After that, the primary antibodies (goat anti‐lipid A LPS polyclonal antibodies, Thermo Fisher Scientific, US) were incubated at 4 °C overnight and then incubated at room temperature with the secondary antibody (Cy3‐ donkey anti‐goat, Beyotime Biotechnology Co., Shanghai, China) for 1 h. F‐actin was stained with ActinGreen 488 ReadyProbes, and the nuclei were counterstained with DAPI (Beyotime Biotechnology Co., Shanghai, China).

For the autophagosome/autophagolysosome experiment, A549 cells were seeded in 1% DMED/F‐12 at a concentration of 3 × 10^4^ cells per well in 24‐well plates. Host cells were infected with GFP‐RFP‐LC3 adenovirus (MOI = 40) (Beyotime Biotechnology Co., Shanghai, China) for 48 h, then cells were infected with OMVs (200 µg mL^−1^) and/or SLAP‐S25 (20 µg mL^−1^) for 6 h, followed by fixation in 4% paraformaldehyde.

For static images, fixed and stained cellular samples were captured by a Leica SP8 confocal microscope. Images were analyzed and merged by LAS AF Lite software (Leica Biosystems, Germany).

### Western Blotting

Western blotting was utilized to analyze the expression of PI3KR1, Atg9A, and LC3 proteins. A549 cells were seeded in 1% DMED/F‐12 at a concentration of 5 × 10^5^ cells per well in 6‐well plates. Cells were then infected with the OMVs (200 µg mL^−1^) and/or SLAP‐S25 (20 µg mL^−1^) at 37 ˚C for 6 h. The primary antibodies included rabbit anti‐ PI3KR1 (ABclonal Technology Co., Wuhan, China), rabbit anti‐Atg9A (Cell Signaling Technology, US), rabbit anti‐LC3 (Sigma, US), and rabbit anti‐*β*‐actin antibodies (MBL International, US). The secondary antibody was a goat anti‐rabbit antibody (Beyotime Biotechnology Co., Shanghai, China). The gray values of protein bands were quantified by ImageJ software.

### Mouse Infection Models

For the mouse lung infection model, C57BL/6 female mice (*n* = 8 per group, one group for hematoxylin and eosin‐stained and immunohistofluorescence) were injected with cyclophosphamide (50 mg kg^−1^) at a specified time. Following anesthesia, mice were intranasally inoculated with 50 µL of *P. aeruginosa* PAO1 (1 × 10^6^ CFUs) per mouse. After 5 h, mice were treated with intraperitoneal administration of different treatments (4 mg kg^−1^, GC, GS, GA, GCS, GCA, GSA, and GCL groups were gelatin‐coated nanogels loaded with colistin, SLAP‐S25, AOAH, colistin+SLAP‐S25, colistin+AOAH, SLAP‐S25+AOAH, and celecoxib, respectively). The bacterial loads, TNF‐*α*, and IL‐6 concentrations in the lungs were counted after 5 d.

For the mouse peritonitis‐sepsis model, C57BL/6 female mice (*n* = 8 per group, one group for hematoxylin and eosin‐stained and immunohistofluorescence) were intraperitoneally injected with *P. aeruginosa* PAO1 (200 µL, 5 × 10^6^ CFUs). After 1 h post‐infection, mice were treated with intraperitoneal administration of different treatments (6 mg kg^−1^). Once the infected mice died, different organs, including the heart, liver, spleen, lung, and kidney, were collected for bacterial counts and cytokine analysis within 48 h. Survival rates were also quantified after 48 h.

### Statistical Analysis

Statistical analysis was performed using GraphPad Prism 8.0 (GraphPad Software, Inc.). A one‐way ANOVA test was used for all statistical analyses. All data were expressed as means ± s.d.

## Conflict of Interest

The authors declare no conflict of interest.

## Supporting information

Supporting Information

## Data Availability

The data that support the findings of this study are available in the supplementary material of this article.
