# Supplementary material for: In Situ Neutralization and Detoxification of LPS to Attenuate Hyperinflammation
Source: Adv Sci (Weinh). 2023 Jul 10;10(26):2302950. doi: 10.1002/advs.202302950 (PMC10502683; doi:10.1002/advs.202302950)
Supplement: Supplementary file 1 — Supporting Information [file ADVS-10-2302950-s001.pdf]

ADVANCED SCIENCE Open Access

Supporting Information

for *Adv. Sci.*, DOI 10.1002/advs.202302950

In Situ Neutralization and Detoxification of LPS to Attenuate Hyperinflammation

Xiaoyu Li, Shaoqi Qu, Xiangbin Song, Congming Wu, Jianzhong Shen and Kui Zhu**

Supporting Information

In situ neutralization and detoxification of LPS to attenuate hyperinflammation

Xiaoyu Li,[†] *Shaoqi Qu,*[†] *Xiangbin Song, Congming Wu, Jianzhong Shen,*[*] *Kui Zhu*[*]

X. Y. Li, Dr. S. Q. Qu, X. B. Song, Prof. C. M. Wu, Prof. J. Z. Shen, Prof. K. Zhu
National Key Laboratory of Veterinary Public Health Security
College of Veterinary Medicine, China Agricultural University
Beijing 100193, China.
Email: sjz@cau.edu.cn (J. S.); zhuk@cau.edu.cn (K. Z.)

Prof. J. Z. Shen, Prof. K. Zhu
Guangdong Laboratory for Lingnan Modern Agriculture, Guangzhou 510642, China

† These authors contributed equally to this work.

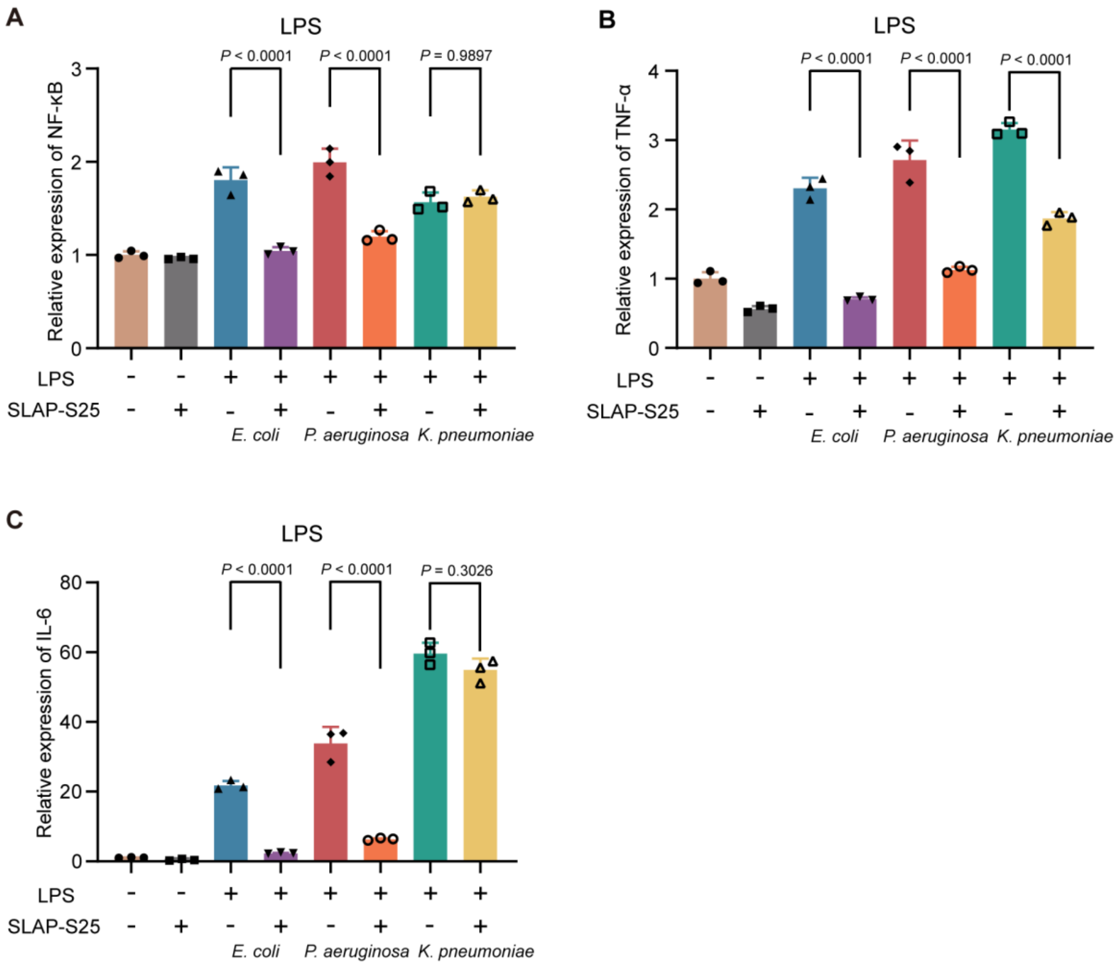

Figure S1. SLAP-S25 attenuates inflammatory response induced by LPS.
A, B, C) Relative expression of NF-κB (A), TNF-α (B) and IL-6 (C) in mouse alveolar macrophages (MH-S) cells in the presence of diverse bacteria (*E. coli*, *P. aeruginosa* and *K. pneumoniae*) based on qRT-PCR.
Experiments in A, B and C were performed as three biologically independent experiments, and the mean ± s.d. is shown, $n = 3$. *P*-values were determined using One-way ANOVA test.

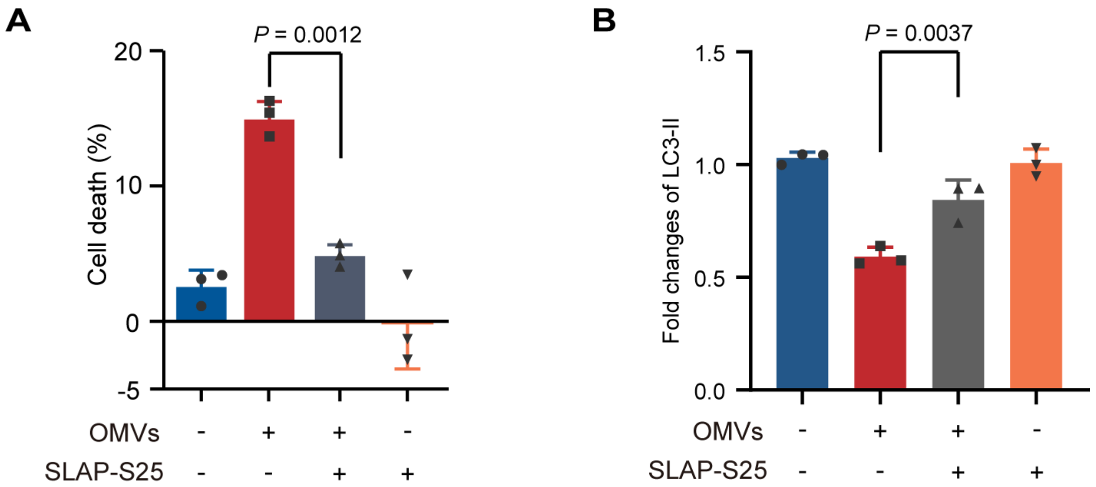

Figure S2. SLAP-S25 attenuates the cell death induced by OMVs derived from *K. pneumoniae*.

A) Cell mortality in A549 cells treated with OMVs and/or SLAP-S25 as determined by lactate dehydrogenase (LDH) assay.

B) Relative expression and quantification of autophagy relative protein LC3-II in A549 cells.

Experiments in A and B were performed as three biologically independent experiments, and the mean ± s.d. is shown, $n = 3$. P-values were determined using One-way ANOVA test.

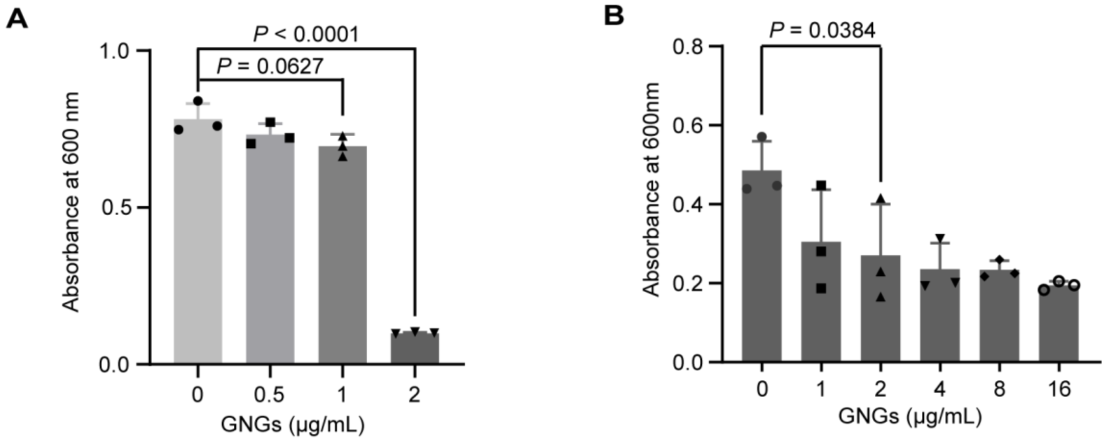

Figure S3. GNGs exert inhibition of bacteria growth and biofilm formation.
A) Bacterial density of *P. aeruginosa* PAO1 in the presence of GNGs for 24 h.
B) Inhibitory effects of GNGs in a dose manner on *P. aeruginosa* biofilm formation. The absorbance was measured at 600 nm using an Infinite M200 Microplate reader (Tecan).
Experiments in A and B were performed as three biologically independent experiments, and the mean ± s.d. is shown, $n = 3$. *P*-values were determined using One-way ANOVA test.

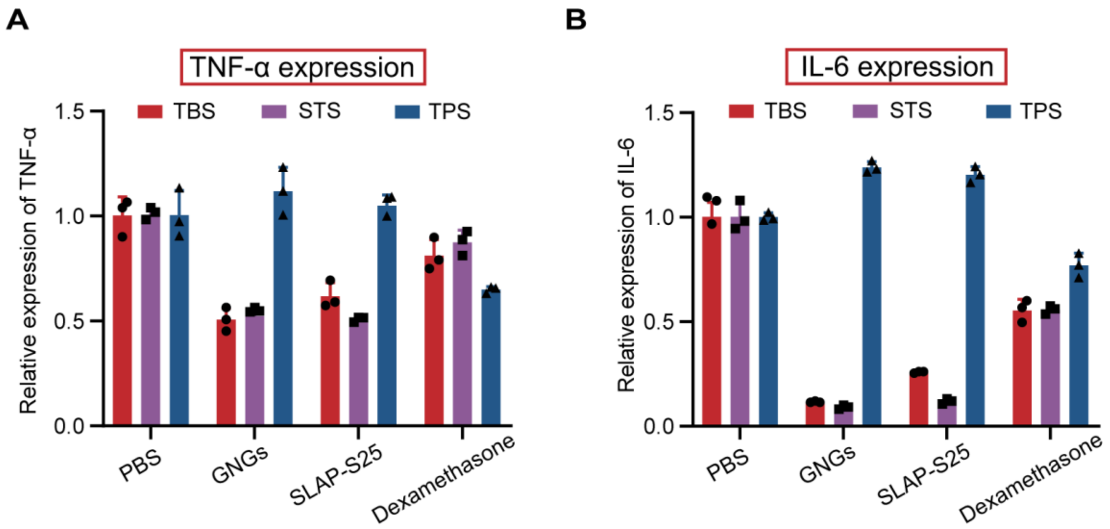

Figure S4. GNGs attenuate the expression of either TNF-α or IL-6 in TBS, STS and TPS.

A, B) Relative expression of TNF-α (A) and IL-6 (B) with the treatments added before (TBS), simultaneous (STS) or after (TPS) the stimulation of LPS in MH-S cells, respectively. Experiments in A and B were performed as three biologically independent experiments, and the mean ± s.d. is shown, $n = 3$.

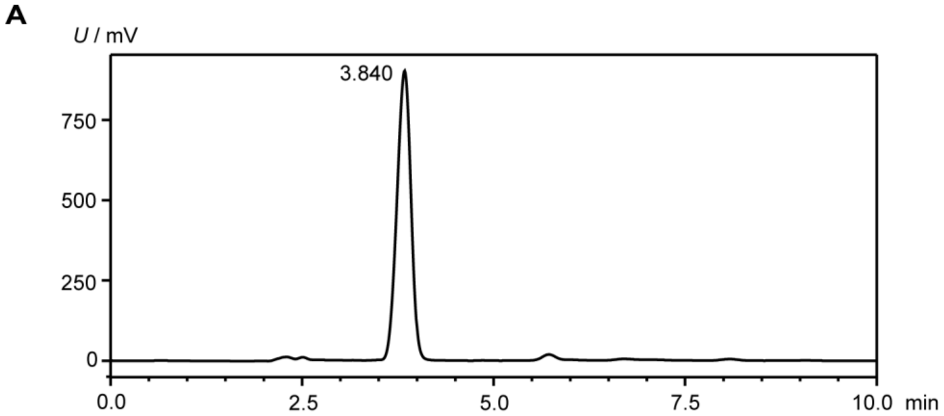

Figure S5. Quantification of prostaglandin using HPLC.

HPLC chromatograms of a prostaglandin standard. The chromatographic separation was carried out on an ODS-C_{18} column, which was kept at a temperature of 30 °C. The mobile phase A (acetonitrile) and mobile phase B (0.02 mol potassium dihydrogen phosphate) at a constant flow rate of 1 mL/min. A sample solution of 20 µL was injected into the HPLC system and detected at a wavelength of 196 nm.

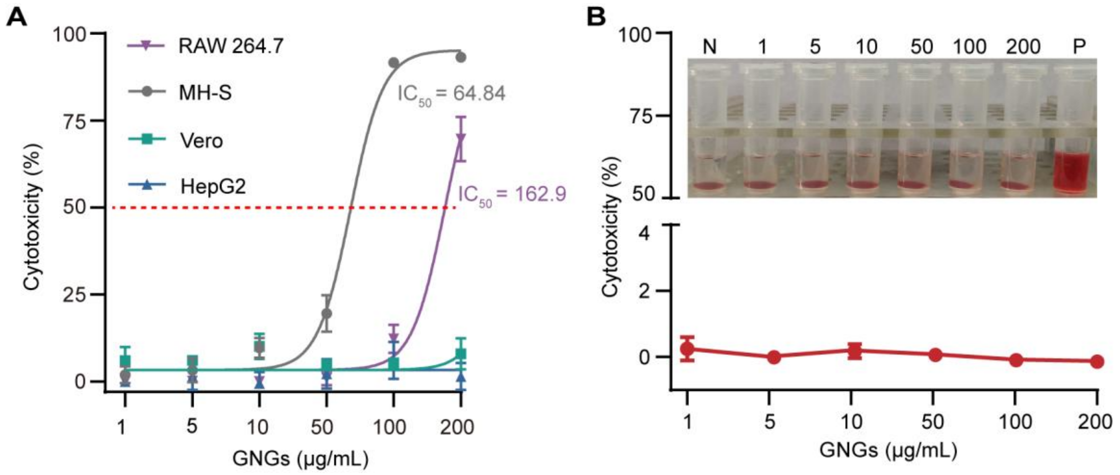

Figure S6. Biocompatibility of GNGs *in vitro*.

A) Cytotoxicity of RAW 264.7, MH-S, Vero, and HepG2 cells treated with GNGs were evaluated using the LDH assay. The result of staurosporine as the positive control was not shown.

B) Hemolytic activity of GNGs to the red blood cells of sheep, N group as the negative control (PBS), P group as the positive control (0.2% Triton X-100).

Experiments in A and B were performed as three biologically independent experiments, and the mean ± s.d. is shown, $n = 3$. P-values were determined using One-way ANOVA test.

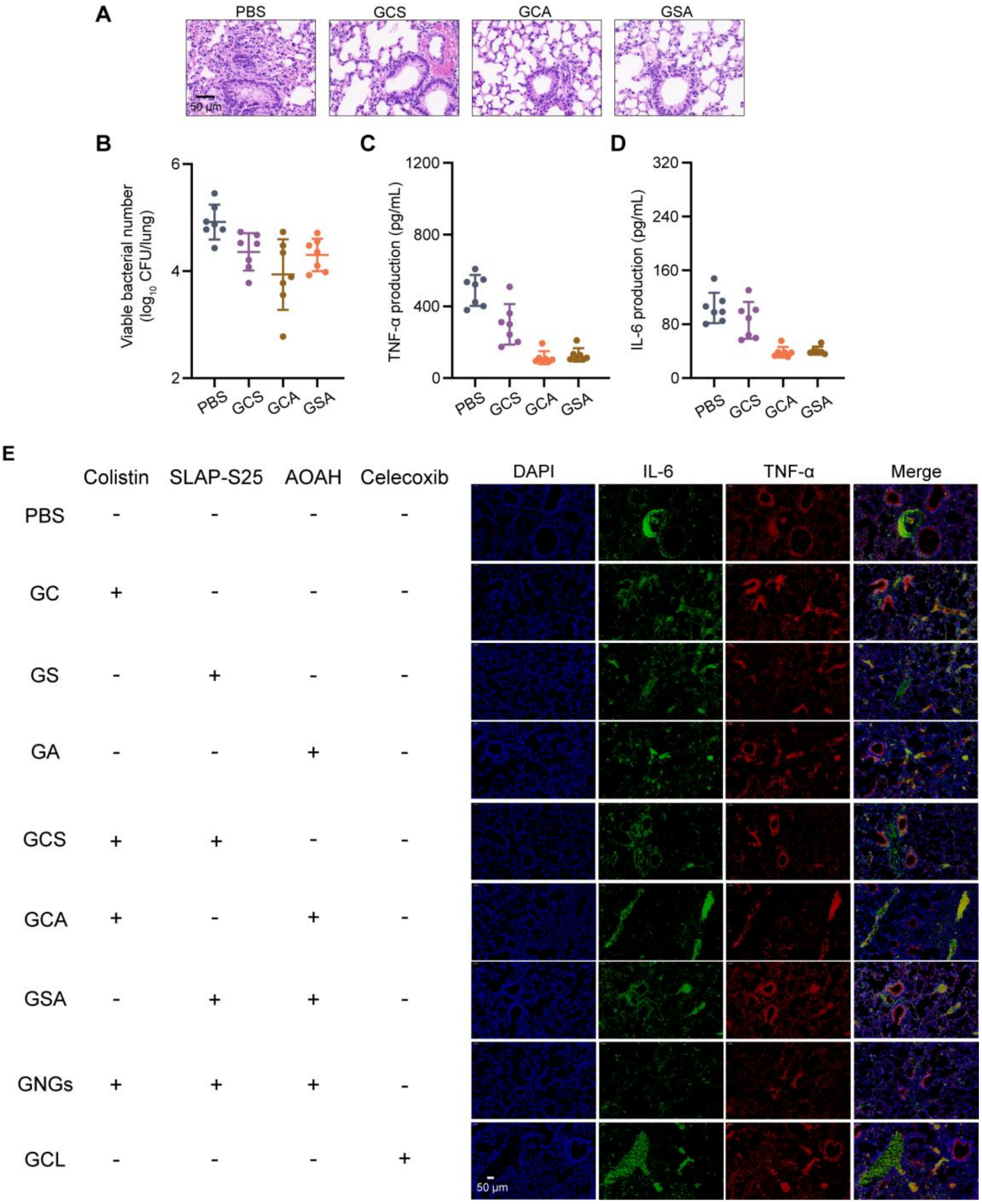

Figure S7. The antibacterial and anti-inflammatory activity in the mouse lung infection model.

Representative hematoxylin and eosin-stained lungs of mice in the lung infection model. Scale bar = 50 μm. B, C, D) Bacterial loads (B), the production of TNF-α (C) and IL-6 (D) in the lungs of mice in the lung infection model. E) Immunohistofluorescence analysis of TNF-α and IL-6 in the lungs of mice in the lung infection model. Scale bar = 50 μm.

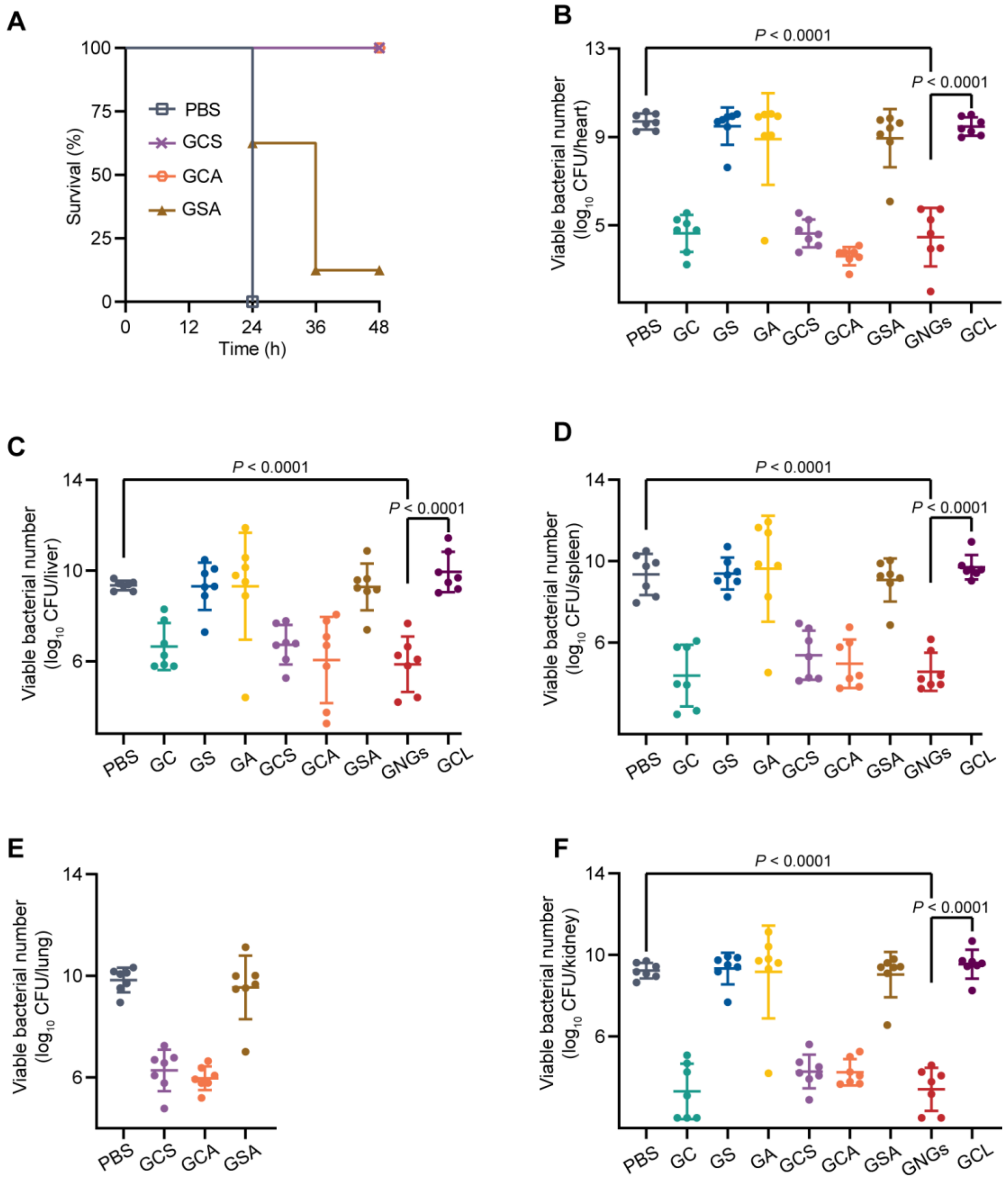

Figure S8. Survival and bacterial loads of mice in the peritonitis-sepsis model.
A) Survival rates of mice in the peritonitis-sepsis model.
B, C, D, E, F) Bacterial loads of mice in major organs includes heart (B), liver (C), spleen (D), lung (E) and kidney (F) in the peritonitis-sepsis model.
P-values were determined using One-way ANOVA test.

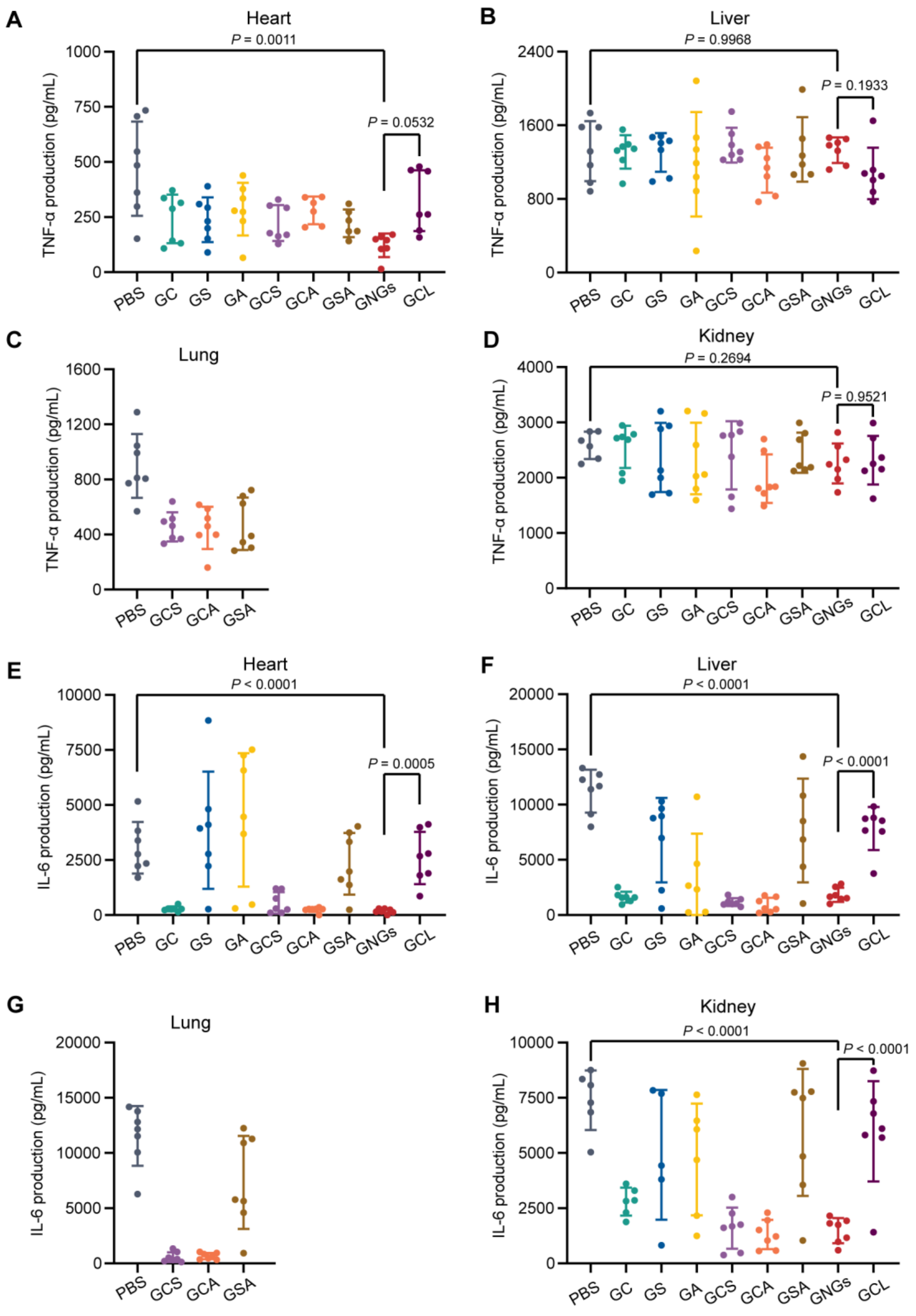

Figure S9. Inflammatory response of mice in the peritonitis-sepsis model.
A, B, C, D) The production of TNF-α in major organs include heart (A), liver (B), lung (C) and kidney (D) in the peritonitis-sepsis model.
E, F, G, H) The production of IL-6 in major organs include heart (E), liver (F), lung (G) and kidney (H) in the peritonitis-sepsis model.
P-values were determined using One-way ANOVA test.

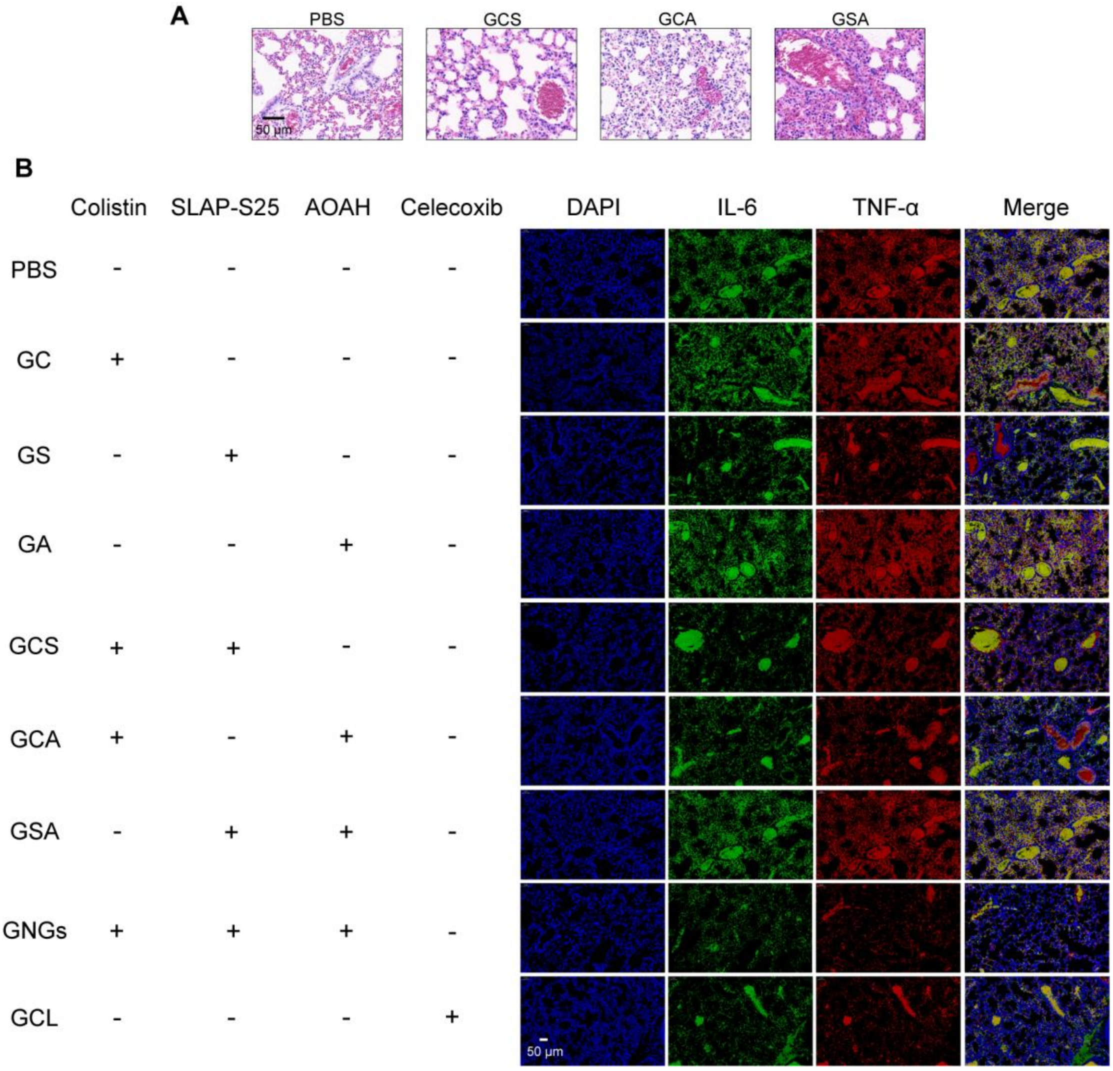

Figure S10. Histological analysis of lung in mice in the peritonitis-sepsis model.
A) Representative hematoxylin and eosin-stained lung histology sections of mice in the peritonitis-sepsis model. Scale bar = 50 μm.
B) Representative images of cytokines in the lungs of mice in the peritonitis-sepsis model. Blue (DAPI, nuclei), green (IL-6), red (TNF-α), and yellow (merged). Scale bar = 50 μm.

Table S1. Comparison of properties of different antimicrobial peptides.

Name	Molecular weight (g/mol)	Affinity to LPS (M-1)	Neutralizing modified LPS	References
SLAP-S25	1229.56	3.10×10^{5}	Yes	[1]
Peptide 3	1851.96	$2.9\pm0.1\times10^{6}$	ND	[2]
I1W	1625.16	3.45×10^{3}	ND	[3]
Pentamidine	340.42	ND	Yes	[4]
Thanatin	2436.30	$9.15\pm0.85\times10^{6}$	ND	[5]
Papiliocin	4002.81	1.58×10^{7}	ND	[6]
Polymyxin B	1203.50	3.04×10^{6}	No	[6]

ND: no data

References

1. M. Song, Y. Liu, X. Huang, S. Ding, Y. Wang, J. Shen, K. Zhu, *Nat. Microbiol.* **2020**, 5, 1040.
2. A. Rustici, M. Velucchi, R. Faggioni, M. Sironi, P. Ghezzi, S. Quataert, B. Green, M. Porro, *Science (New York, N.Y.)* **1993**, 259, 361.
3. D. Shang, Q. Zhang, W. Dong, H. Liang, X. Bi, *Acta Biomater.* **2016**, 33, 153.
4. J. M. Stokes, C. R. MacNair, B. Ilyas, S. French, J. P. Cote, C. Bouwman, M. A. Farha, A. O. Sieron, C. Whitfield, B. K. Coombes, E. D. Brown, *Nat. Microbiol.* **2017**, 2, 17028.
5. B. Ma, C. Fang, L. Lu, M. Wang, X. Xue, Y. Zhou, M. Li, Y. Hu, X. Luo, Z. Hou, *Nat. Commun.* **2019***,* 10, 3517.
6. M. Krishnan, J. Choi, A. Jang, S. Choi, J. Yeon, M. Jang, Y. Lee, K. Son, S. Y. Shin, M. S. Jeong, Y. Kim, *Proc. Natl. Acad. Sci. U.S.A.* **2022**, 119, e2115669119.
